# Recent Developments in Antibody-Based Assays for the Detection of Bacterial Toxins

**DOI:** 10.3390/toxins6041325

**Published:** 2014-04-11

**Authors:** Kui Zhu, Richard Dietrich, Andrea Didier, Dominik Doyscher, Erwin Märtlbauer

**Affiliations:** Institute of Food Science, Department of Veterinary Sciences, Ludwig-Maximilians-University Munich, Oberschleißheim 85764, Germany; E-Mails: zhukcau@gmail.com (K.Z.); r.dietrich@mh.vetmed.uni-muenchen.de (R.D.); a.didier@mh.vetmed.uni-muenchen.de (A.D.); dominik.doyscher@mh.vetmed.uni-muenchen.de (D.D.)

**Keywords:** bacterial toxins, antibodies, immunoassay, nanomaterials, microfluidics

## Abstract

Considering the urgent demand for rapid and accurate determination of bacterial toxins and the recent promising developments in nanotechnology and microfluidics, this review summarizes new achievements of the past five years. Firstly, bacterial toxins will be categorized according to their antibody binding properties into low and high molecular weight compounds. Secondly, the types of antibodies and new techniques for producing antibodies are discussed, including poly- and mono-clonal antibodies, single-chain variable fragments (scFv), as well as heavy-chain and recombinant antibodies. Thirdly, the use of different nanomaterials, such as gold nanoparticles (AuNPs), magnetic nanoparticles (MNPs), quantum dots (QDs) and carbon nanomaterials (graphene and carbon nanotube), for labeling antibodies and toxins or for readout techniques will be summarized. Fourthly, microscale analysis or minimized devices, for example microfluidics or lab-on-a-chip (LOC), which have attracted increasing attention in combination with immunoassays for the robust detection or point-of-care testing (POCT), will be reviewed. Finally, some new materials and analytical strategies, which might be promising for analyzing toxins in the near future, will be shortly introduced.

## 1. Introduction

**Figure 1:**
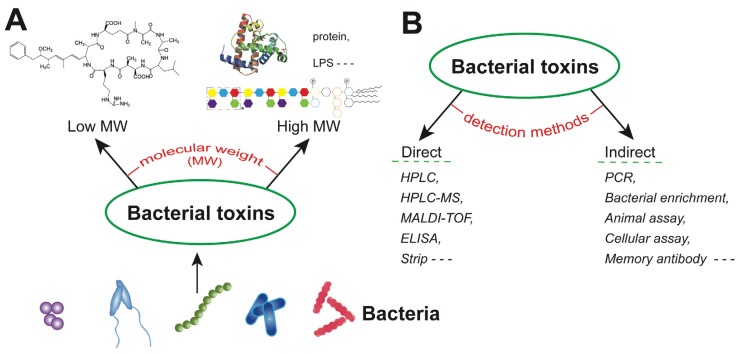
(**A**) Schematic representation of bacterial toxins of low and high molecular weight (MW); and (**B**) examples for direct or indirect detection methods of bacterial toxins. LPS: lipopolysaccharides; HPLC: high-performance liquid chromatography; HPLC-MS: HPLC-mass spectrometry; MALDI-TOF: matrix-assisted laser desorption ionization time-of-flight; ELISA: enzyme-linked immunosorbent assay; and PCR: polymerase chain reaction.

To avoid potential hazards for human and animal health, accurate and reliable analysis of bacterial toxins is critical in clinical diagnostics, food analysis, water monitoring, as well as for bio-security/defense purposes. Bacterial toxins are generally catalogued into exotoxins (peptides and proteins) produced by both Gram-positive and Gram-negative bacterial pathogens and endotoxins (lipopolysaccharides, LPS) produced by Gram-negative bacteria. These toxins cover a broad range of molecular weights, from less than 1000 Da to more than 100,000 Da (Figure 1A), exhibit different physico-chemical properties and cause a broad variety of clinical symptoms, ranging from mild diarrhea and emesis to severe and fatal neurological disorders [1,2,3,4]. According to their preferred targets, bacterial exotoxins may be grouped into toxins acting at the host cell surface and intracellularly active toxins [5]. Pore forming proteins [6,7] and AB-type of toxins (usually consisting of one A- and several B-subunits) [8,9] represent classical examples of the respective groups.

To achieve the selective and sensitive detection of bacterial toxins, many methods have been developed in the past decade, covering direct and indirect approaches (Figure 1B). On the one hand, the toxins are directly captured by antibodies, or measured by mass spectrometry. On the other hand, the functional properties of the toxins are used to trigger effects in animals or cells; also, the bacterial species or the genes involved in toxin production may be detected. These indirect assays can provide valuable information, particularly when searching for unknown bacterial toxins or when direct methods are not available. Direct instrumental assays rely on sophisticated equipment, such as high-performance liquid chromatography (HPLC), HPLC with tandem mass spectrometry (HPLC-MS/MS) and matrix-assisted laser desorption ionization time-of-flight mass spectrometry (MALDI-TOF MS) to decipher the toxin profile [10,11,12]. Detection of bacterial toxins by antibody based assays or immunoassays has also been a successful approach for decades and still gains much attention due to the inherent advantages, such as simplicity, speed and cost-effectiveness. This review is focused on antibody-based techniques for the detection of bacterial toxins.

## 2. Antibodies and Immunoassays

**Figure 2:**
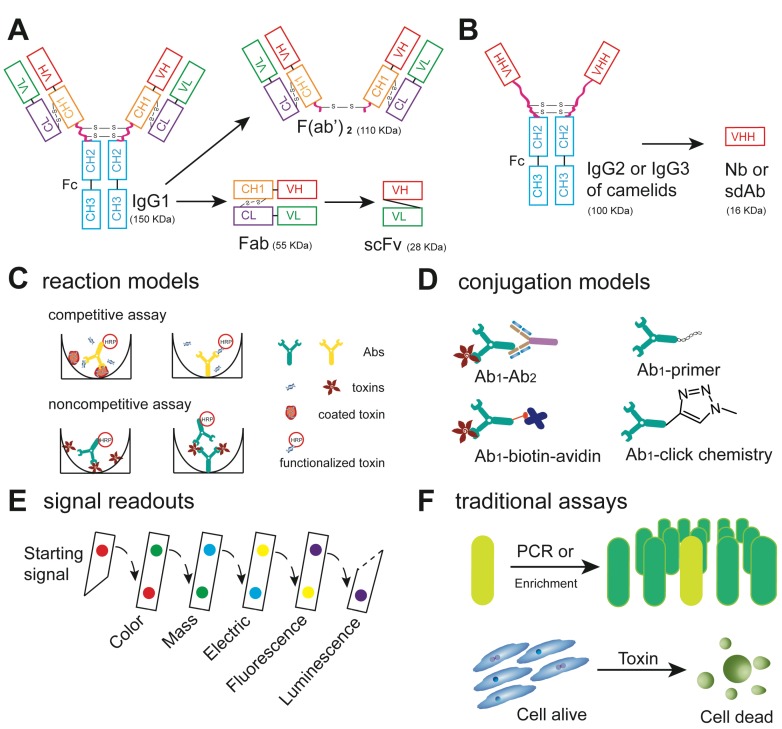
Antibodies and assays: (**A**) types of antibodies used for immunoassay, immunoglobulin G (IgG) and related fragments (VL: variable light chain; VH: variable heavy chain; CL: constant light chain; CH: constant heavy chain; Fab: antigen binding fragment; and scFv: single-chain variable fragment); (**B**) heavy chain antibody (hcAb) and related fragments (VHH: variable domain of heavy chain antibodies; Nb: nanobody; and sdAb: single domain antibody); (**C**) the recognition of toxin targets by antibodies in competitive and noncompetitive assays; (**D**) labeling of primary antibodies for signal generation (Ab: antibody); (**E**) common readout techniques involved in immunoassays; and (**F**) examples for indirect assays. The amplification of toxin genes was done by PCR, the enrichment culture of the toxin producing organism and functional assays, such as cytotoxicity testing on mammalian cells.

The core principle of immunoassays is the specific recognition of the target of interest by an antibody, which is the key component in any test. Several types of antibodies have been introduced for antibody-based sensing of bacterial toxins [13]. Antibodies (Figure 2A) represent a group of glycoproteins possessing two distinct types of polypeptide chains. Both the light chain and the heavy chain show a variable region of the heavy (VH) and light (VL) chains at their amino terminal end, whereas the remaining part of the polypeptide chain is referred to as the constant region (constant heavy (CH) and constant light (CL) chain). The variable regions of both chains contain a hypervariable part, which represents the antigen binding site (antibody combining site) or “paratope”. After the folding and combining of the light and heavy chains, this hypervariable region of the immunoglobulin shows a structure complementary to the corresponding part of the antigen molecule, which is referred to as the antigenic determinant or “epitope”. The antibodies produced in an animal species are polyclonal in nature (polyclonal antibody, pAb) and synthesized and secreted by plasma cells, derived from different B-lymphocytes. These lymphocytes may be fused with myeloma cells. The myeloma cells provide the genes for continued cell division, whereas the lymphocytes provide the functional immunoglobulin genes. The fused cells are called hybrid cells or hybridomas, and each hybridoma produces identical copies of one antibody, *i.e.*, monoclonal antibodies (mAb). For immunoassays, preferably antibodies of the immunoglobulin G (IgG) class are employed. Whereas each mAb recognizes a single epitope, pAbs may bind to a range of epitopes present on the antigen used for the immunization. For specific applications, enzymes, such as papain and pepsin, are used to digest the intact antibody to generate small antigen binding fragments [14]. A new approach to generate half antibody fragments for biosensor applications by reduction via tris(2-carboxyethyl) phosphine (TCEP) was described recently [15]. Alternatively, recombinant techniques are used to produce the single-chain variable fragment (scFv) containing the variable regions of the VH and VL chains of the original IgG linked by a short peptide [16]. Recombinant antibodies and antibody fragments show distinct advantages to improve the sensitivity of biosensor applications, since they facilitate the loading of more and properly orientated antigen binding fragments onto a limited surface area [17]. Furthermore, the recently described technique of the direct pairing of two independent antibody specificities to create new bispecific antibodies [18,19] may enable the enhanced detection of bacterial toxins in the future.

An alternative to conventional antibodies, heavy chain antibodies (hcAbs, Figure 2B), found in camelids, have gained considerable attention, because of their unique structure, being composed of heavy chains only [20]. Fragments of hcAb, named nanobody (Nb) or single domain antibody (sdAb), have already been used for the detection of bacterial toxins, such as cholera toxin (CT) [21]. Furthermore, the design and production of recombinant Nbs from hcAbs is simplified, because only the variable region of a single (heavy) chain must be cloned and expressed in *E. coli* or other organisms [22].

Immunoassays include several steps: (i) the recognition of toxin targets by antibodies; (ii) subsequent signal transduction; and (iii) readout techniques providing qualitative or quantitative results. Competitive and noncompetitive assays may be employed in the first step, depending on the number of epitopes available on the toxins (Figure 2C). Competitive methods are based on the competition of free and labeled (functionalized) or solid phase-bound antigens for a limited number of antibody combining sites. In most cases, the assay response represents the bound labeled antigen and is therefore inversely proportional to the concentration of the free antigen. This type of assay is used for the detection of low molecular weight toxins, such as the monocyclic heptapeptide, microcystin, produced by Cyanobacteria, which have only one epitope. Two variants of noncompetitive assays may be used to detect bacterial protein toxins. The so-called sandwich enzyme immunoassay can only be used for the detection of macromolecules, such as protein toxins, having at least two antigenic determinants in suitable steric positions, enabling two antibodies (capture and detection antibody) to bind to the antigen. In the second variant, the solid phase is coated directly with the toxin, and the amount of toxin bound is determined using specific labeled antibodies. In both cases, the assay response is directly proportional to the concentration of the target antigen.

In the second step, different protocols can be used to generate the final readout after primary antibody binding. To permit the sensitive observation of the antigen-antibody reaction, antigens or antibodies have to be labeled either directly or indirectly. Protocols for indirect labeling include functionalized secondary antibodies and the biotin-avidin system to bridge the antigen-antibody reaction and signal generation (Figure 2D) [23,24]. Direct modification of the primary antibody can be achieved by biomolecules, such as horseradish peroxidase (HRP) or alkaline phosphatase (ALP), and may result in decreased affinity and stability induced by unspecific side effects of the coupling chemistry and/or steric hindrance by the attachment of the reporter enzymes. Recently, oligonucleotide-modified primary antibodies have been implemented in immuno-PCR methods to detect Shiga toxin 2 (Stx2) and Stx2 variants [25]. However, the low efficiency of the preparation of the chimera has hindered immuno-PCR from wide acceptance [26]. Alternatively, polymer and “click” chemistry may be useful ways to improve the labeling of the primary antibody. For example, more enzymes can be anchored on the surface of stretch polymers to increase the ratio of enzyme to antibody [27]. Compared to the noncovalent binding involved in protocols utilizing secondary antibodies or biotin-avidin, covalent coupling using “click” chemistry offers several advantages. “Click” chemistry was first described for chemical reactions yielding high amounts of specifically and quickly joined small units; one of the most popular reactions is the azide-alkyne, cycloaddition, with or without catalysis by copper [28,29].

In the third step, the final readout is generated. Although label-free methods, such as surface plasmon resonance (SPR) and electrochemical sensors, have been used for the detection of CT and the LPS of Gram-negative bacteria with high sensitivity [30,31,32], the vast majority of immunoassays utilize labeled immunoreagents. The signal can be amplified by enzymes, which are widely used for colorimetry-based qualitative and quantitative assays. However, the detection of trace amounts of toxin often requires further signal enhancement, and many other methods, such as fluorescence, luminescence, electronic signal and mass spectrometry, have been employed to improve the sensitivity (Figure 2E). In the following section, we will summarize how these approaches enable signal amplification, with special emphasis on the use of nanomaterials.

## 3. Nanomaterials for Immunoassays

As one of the most innovative and attractive technologies, nanotechnology has also entered bioanalysis. Diverse nanomaterials of different sizes, shapes and functional properties have been constructed. Herein, we will focus on the ones that have already been used for the detection of bacterial toxins in the past few years and on those that have the potential to serve this task. Nanomaterials can be used directly for different readouts or indirectly as carriers or anchored supports for other labels, such as enzymes and fluorescent probes.

### 3.1. Gold Nanoparticles (AuNPs)

AuNPs have had a long history of application in on-site tests since their first use as a colorimetric readout in lateral flow immunoassays (or strips), such as home pregnancy tests. Several recent reviews focus on the synthesis and properties of AuNPs [33,34,35]. In the following part, we will concentrate on recent applications of AuNPs for the detection of bacterial toxins and on the mechanisms behind the assays.

Optical immunoassays are based on direct color generation mediated by AuNPs either on solid surfaces or in solution. Lateral flow immunoassay, for example, is typically performed on hydrophobic nitrocellulose or cellulose acetate membranes. In sandwich-type assays, the red test line reports the presence of the target of interest, due to the accumulation of AuNP-labeled detection antibodies in the test zone. Recently, a dual assay of this type for the simultaneous detection of botulinum neurotoxin serotype A and B on a single strip has been developed [36]. In solution, after the aggregation of AuNPs through an unspecific electrostatic interaction or after specific binding by antibodies, a dramatic shift from red (dispersed particles) to blue (aggregated particles) color is observed, which can be used for visual detection or quantification. This phenomenon is based on localized surface plasmon resonance (LSPR), which is caused by the interaction between AuNPs and the incident light of a larger wavelength than the diameter of the AuNPs [37]. The cholera toxin B subunit (CTB), for example, can selectively bind to AuNPs modified by a lactose derivative and induce the aggregation of AuNPs, allowing the detection of 54 nM (3 µg mL^−1^) of toxin [38].

**Figure 3:**
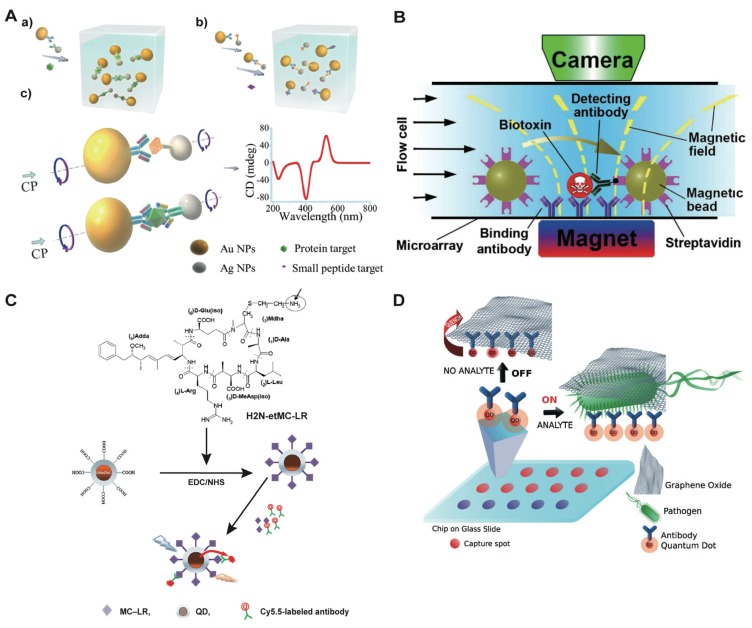
(**A**) Schemes of gold and silver hetero dimer-based chiroplasmonic methods for (**a**) assaying proteins; (**b**) microcystin-LR. Reprinted with permission from [39]. Copyright 2013 American Chemical Society; (**B**) Scheme of streptavidin functionalized magnetic nanoparticles (MNPs) for the simultaneous detection of five bacterial toxins in a microarray. Reprinted with permission from [24]. Copyright 2013 American Chemical Society; (**C**) Scheme of indirect competitive immunoassay for the detection of microcystin-LR based on the conjugation of quantum dots (QDs) and aminoethyl microcystin-LR. Reprinted with permission from [44]. Copyright 2014 Elsevier; (**D**) Scheme of Ab-QD-based microarrays for pathogen detection. In the presence of captured pathogen by the Ab-QD probes, the fluorescence of QDs are in the ON state, whereas in the absence ofthe pathogen, QDs are quenched by fluorescence resonance energy transfer (FRET) interaction between QDs and graphene oxide (GO) (OFF state). Reprinted with permission from [45]. Copyright 2013 WILEY-VCH Verlag GmbH & Co. KGaA, Weinheim. CP: chiroplasmon; NPs: nanoparticles; EDC: 1-ethyl-3-(3-dimethylaminopropyl)carbodiimid; NHS: N-hydroxysuccinimide; and CD: circular dichroism.

A recent exciting example is the chiroplasmonic immunoassay for the detection of microcystin-LR [39] (Figure 3A). Taking advantage of the chiroplasmonic properties of heterodimers of AuNPs and silver nanoparticles (AgNPs) bridged by antibody-antigen complexes, 8 × 10^−13^ M of microcystin-LR could be detected by circular dichroism (CD) measurement in a competitive assay. This is probably the lowest detection limit achieved up to now for this toxin, and this novel assay holds great promise also for the sensitive analysis of high molecular weight bacterial toxins, such as protein toxins or LPS in sandwich-type assays. Although not used for the detection of toxins so far, a new plasmonic enzyme-linked immunosorbent assay (ELISA) should be mentioned. Instead of color generation by the reporter enzyme, enzymatic activity is used to grow AuNP complexes with an ill-defined morphology, but exhibiting an intense blue color [40]. The authors choose a sandwich ELISA model for the ultrasensitive detection of two protein markers, which could be easily adapted as an alternative protocol for the detection of bacterial toxins.

The distinctive electronic properties of AuNPs can also be used for electrochemical signal amplification [31]. The fabrication of electrodes implementing AuNPs functionalized with antibodies has considerably improved the selectivity and sensitivity of different electrochemical methods. An electrochemical ELISA platform for the sensitive detection of anthrax protective antigen (PA) utilized nanostructured gold electrodes coated with a binding peptide for the specific capture of PA [41]. The assay exhibits a limit of detection of less than 3 pM, when tested with serum samples. At this point, we would like to mention that beside the detection of toxins, numerous alternative assays based on antibody functionalized AuNPs have been developed for the precise and rapid detection of bacteria. Mass spectrometry-based immunosensors using antibody modified AuNPs have been constructed for the quantification of *E. coli*, *Staphylococcus aureus* and *Salmonella enterica* [42]. Furthermore, for the detection of *Bacillus cereus,* an amperometric immunosensor decorated with AuNPs coated with mAbs against of *B. cereus* was established, exhibiting an extremely low limit of detection of 10 colony forming units (CFU) mL^−1^ [43].

### 3.2. Magnetic Nanoparticles (MNPs)

MNPs or magnetic beads (MB), especially iron oxides (Fe_3_O_4_), have attracted considerable attention in biomedical and bioanalytical fields [46]. MNPs can be easily separated or enriched from complicated matrices by simply applying a magnetic field. Based on this phenomenon, different functionalized MNPs have been used for the specific or nonspecific capture and detection of bacteria and related products [47,48,49]. For example, a conductometric immunosensor has been developed for the generic and rapid detection of Gram-negative bacteria [31]. The combination of MNPs functionalized with antibodies for capture and modified AuNPs for detection is widely used for the detection of bacteria [47,50]. The adaption of this method will not only facilitate the enrichment of bacterial toxins at low concentrations from complex sample matrices, but also enable simple readouts, due to the unique properties of the MNPs.

Furthermore, based on the dramatic progress made in the synthesis of nanomaterials, new multiple functional core-shell-structured particles consisting of an MNP core and Au or silicon functionalized shells have been prepared [46]. For instance, gentamicin-modified fluorescent MNPs with Fe_3_O_4_ cores and fluorescent silica (SiO_2_) shells were synthesized to capture Gram-negative bacteria [51]. Furthermore, magnetic silica NPs functionalized with antibodies via “click chemistry” have been reported for the detection of microcystin-LR [52]. Most interestingly, MNPs can also be used directly as the readout for the detection of bacterial toxins. Five bacterial toxins, including CT, heat-labile toxin from *E. coli*, enterotoxins A and B and toxic shock syndrome toxin (TSST) from *S. aureus* were simultaneously detected in an immunoarray for water, meat and milk samples [24] (Figure 3B). Briefly, the toxins are captured by specific antibodies coated on a microarray and then labeled with biotinylated detection antibodies. The assay signal is generated by scanning the microarray surface with streptavidin-coated MB in a shear flow. This ultrasensitive assay detects 0.1 to 1 pg mL^−1^ of toxin in less than 10 min, *i.e.*, 100 zeptomoles or 100,000 molecules of CT in a sample volume of 0.1 mL. Furthermore, nonlinear magnetization of MNPs has been used for a novel sandwich immunoassay on a 3D fiber solid phase for the detection of Staphylococcal Enterotoxin A (SEA) and TSST in milk samples [53]. This label-free assay achieves a limit of detection as low as 4 pg mL^−1^ and 20 pg mL^−^^1^ for TSST and SEA, respectively. Antibodies coupled to fluorescent magnetic microspheres allowed the multiplex toxin detection of botulinum neurotoxins type A and B and Staphylococcal Enterotoxin B (SEB) and other toxins in a suspension assay [54].

Besides the application of MNPs for sample separation, biological imaging, drug delivery, magnetic sensors and conjugation with enzymes for signal simplification [46,55], Fe_3_O_4_ MNPs exhibit intrinsic peroxidase-like activity, which was discovered in 2007 [56]. The enzymatic activity, similar to natural peroxidases, was used to develop novel immunoassays with antibody-modified MNPs for the detection of protein targets in both direct and sandwich formats, integrating three functions (capture, separation and detection). Compared to natural enzymes, such as HRP, MNPs are more stable, easy-to-produce, multi-functional and inexpensive. The enzymatic activity of Fe_3_O_4 _MNPs has been used in immunoassays and PCR [57,58] and may also be used as an alternative label for the detection of bacterial toxins, and robust assays could be constructed utilizing multifunctional MNPs.

### 3.3. Quantum Dots (QDs)

QDs are semiconductor nanocrystals that have been widely used in many fields, firstly introduced as fluorescent probes for biomedical applications by two independent labs [59,60]. Compared to the traditional chemical fluorescent dyes and proteins, QDs have shown several advantages, such as broad excitation spectra, high quantum yield, large Stokes shifts and high photostability [61]. There are numerous applications of QDs for the detection of bacterial toxins [62]. For example, a fluorescent sandwich immunoassay for the quantification of botulinum neurotoxin serotype A (BoNT/A) employed QDs functionalized with high affinity antibodies for detection [63]. Furthermore, an indirect competitive immunoassay was built for the analysis of microcystin-LR in water [44]. The carboxyl-coated QDs were coupled with aminoethyl-microcystin-LR and used as donors together with Cy5.5-labeled antibodies as receptors to construct a fluorescence resonance energy transfer (FRET) system for the sensitive and rapid detection of microcystin-LR in water samples in a portable optofluidic platform (Figure 3C). FRET or related phenomena are routine methods for the detection of ions, small molecules, proteins or nucleic acids [61,62,64]. For example, another FRET assay has been constructed for the detection of microcystin-LR by monoclonal antibody-coated CdSe-CdS core-shell structured QDs [65]. The fluorescence intensity of QDs is quenched in the presence of microcystin-LR, enabling the detection of 6.9 × 10^−11^ mol L^−1^ of toxin in water samples.

A unique property of QDs is that they share a broad excitation spectrum, but can emit different narrow spectra, due to the quantum confinement effect [66]. QDs of a different size can be excited by a single wavelength and generate symmetrical emission bands at different wavelengths, exhibiting large Stokes shifts. This allows the detection of multiple targets in a single assay at the same time [64,67]. As the first example, CdSe-ZnS core-shell structured QDs of different sizes coated with antibodies were used for the simultaneous detection of CT, ricin, shiga-like toxin 1 and SEB in a microtiter plate [68]. The wells of the microtiter plate were coated with different capture antibodies, followed by the addition of the toxin mixture and the four corresponding antibody-QDs conjugates. After excitation at 330 nm, the emitted fluorescence was recorded at 510, 555, 590 and 610 nm for the individual QDs. However, the assay showed major drawbacks, such as insufficient sensitivity and high background signals. Later, a waveguide-based immunosensor platform was developed for the analysis of a PA and lethal factor from *Bacillus anthracis* using QDs as the fluorescent reporters [69]. This assay incorporates multichannel waveguides, enabling the simultaneous detection of the PA (QDs, 605 nm) and lethal factor (QDs, 655 nm) together with an internal standard (QDs, 565 nm) implemented for the optimization of the assay variability. As low as 1 pM of PA and lethal factor, respectively, could be detected in sera.

Progress in surface chemistry has further broadened the spectrum of applications of QDs for bioanalytical purposes. For instance, a new series of zwitterionic ligands with enhanced affinity to the surface of CdSe-ZnS QD and the ability to increase water solubility has been synthesized [70]. Such functionalized QDs retain their optical properties and possess a remarkable stability under harsh conditions. Furthermore, a new method has been reported to produce monovalent QDs by “steric exclusion”. A polymer of phosphorothioate DNA with a defined sequence and length is used to treat commercially available CdSe-ZnS QDs [71]. After passivation by polyethylene glycol (PEG) ligands, the phosphorothioate DNA-wrapped QDs show excellent colloidal and optical properties for versatile applications. It can be envisioned that these new developments could become useful for the detection of bacterial toxins in the near future.

### 3.4. Carbon Nanomaterials

Carbon nanomaterials have attracted enormous interest in nanotechnology, covering a wide range of applications, from biosensing to drug delivery. Compared to metal-derived nanomaterials, such as AuNPs or cadmium-based fluorescent QDs, carbon nanomaterials show good biocompatibility and are environmental friendly [72]. The most common carbon nanomaterials include fullerenes, carbon nanotubes (CNTs), graphene, carbon dots, nanodiamonds and carbon nanofibers. The distinctive optical and electrical properties of carbon nanomaterials make them good candidates for analytical tasks. For example, different carbon-based nanomaterials have been used as adsorbents for sample preparation, particularly for the nonspecific detection of ions, small molecules and bacterial pathogens [73,74]. With the goal of the ultrasensitive detection of bacterial toxins, specific binding interactions based on CNTs and graphene functionalized by specific antibodies will be summarized here.

According to their intrinsic structures, CNTs can be catalogued into single-walled carbon nanotubes (SWNTs) and multiwalled carbon nanotubes (MWNTs) [75]. CNTs have been directly used for the label-free detection of epsilon toxin from *Clostridium perfringens* with a detection limit of about 2 nM and the genomic DNA of Shiga-toxin from *E. coli* [76,77]. Furthermore, CNTs modified with plastic antibodies, lactose and peptides were developed for the detection of microcystins, CT and anthrax PA toxin, respectively [78,79,80]. An electrochemical immunoassay using antibodies immobilized on modified MWNTs was constructed for the sensitive detection of CT [81]. CT first binds to the antibody-coated CNTs and then forms a sandwich complex with cell membrane ligand ganglioside (GM1) functionalized liposomes, from which an electroactive marker is released after treatment with Triton X-100. This assay provides a platform for the ultratrace level detection of CT in a range from 1 × 10^−14^ g mL^−1^ to 1 × 10^−7^ g mL^−1^. In addition, a rapid, simple and sensitive electrical sensor has been proposed using paper impregnated with SWNTs and antibodies against microcystin LR [82]. This assay relies on the formation of antibody-microcystin LR complexes between CNTs, forming a dense percolation network exhibiting a change in conductivity depending on the presence of the analyte. The performance of this rapid assay is similar to that of ELISA, with a detection limit of 0.6 ng mL^−1^. It should be mentioned here that, similar to the catalytic activity of MNPs, both CNTs and carboxyl-modified graphene oxides possess intrinsic peroxidase-like activity [75], although there is no report on the analysis of bacterial toxins based on this phenomenon.

Since the first report on electronic properties of graphene in 2004, a variety of applications have been published [73]. For instance, graphene and chitosan were immobilized on electrodes for the detection of microcystin LR, and antibody-carbon nanosphere-HRP conjugates were used for signal amplification [83]. This approach provides a detection limit of 0.016 μg L^−1^ of microcystin LR in environmental water samples. Graphene oxide (GO), a promising material with superior properties, has been widely used for biomedical applications and analytical purposes [73,75,84,85]. During the process of preparation, GO gains fluorescent properties, a phenomenon that has been directly used to measure microcystins. Antibodies adsorbed on GO sheets specifically capture microcystins attached on AuNPs, which quench the fluorescence of GO by FRET between GO and AuNPs [86]. With the help of broad-spectrum antibodies for microcystins, 0.5 μg L^−1^ and 0.3 μg L^−1^ of microcystin-LR and microcystin-RR, respectively, could be detected. Furthermore, GOs are widely used as fluorescent quenchers to construct different platforms for biosensing. A FRET system that uses an antibody-CdSe/ZnS QD microarray for the detection of *E. coli* O157:H7 has been reported, with QDs as acceptors of energy transfer [45]. GO interacts with QDs through π–π stacking and quenches the fluorescence in the absence of bacteria, while QDs turn on the fluorescence in the presence of bacteria (Figure 3D). Moreover, GO has been used as a scaffold for the enhanced loading of antibodies, enzymes or other biomolecules onto its large surface. For example, for the detection of *Clostridium difficile* toxin B, multiple HRP and HRP-secondary antibodies were conjugated to GO to amplify the test signal, allowing the detection of 0.7 pg toxin mL^−1^ [87].

## 4. Micro Total Analysis Systems (μTAS)

μTAS, known as lab(oratories)-on-a-chip (LOC) and microfluidic devices, have attracted a lot of attention. These systems integrate chemical and biological labs onto miniaturized chips of the centimeter-scale. The integrated chips can perform sophisticated functions, such as sample separation, signal amplification and detection, to produce “sample-in and answer-out” systems [88]. Excellent recent reviews were focused on design and fabrication of chips [88], as well as the application in immunoassays for point of care (POC) diagnostics [89]. Furthermore, one review summarized the detection of pathogens, including viruses, bacteria and bacterial toxins, using LOC devices [90]. In this section, we will focus on recent achievements in antibody-based microfluidic systems and highlight the combination with nanomaterials for the detection of bacterial toxins. Generally, the assays performed in microfluidic platforms may be categorized into: (i) chips designed for the miniaturization of traditional assays, e.g., ELISA [91,92]; and (ii) chips integrating sample pretreatment, signal amplification and readout techniques [93,94,95,96,97]. For example, sandwich and competitive ELISAs were combined with a microfluidic device for single-cell studies to reliably identify intracellular proteins and metabolites [98]. This platform contains all steps for single-cell analysis from cell lysis to ELISA, including incubation periods, repeated washing steps and fluorescent readouts. Another advantage is that the integration of analyte preconcentration on chips can significantly improve detection limits and improve signal-to-noise ratios. For example, a microfluidic ELISA employed a semipermeable membrane for the preconcentration of the target analyte by electrokinetic means [99]. The local concentration of the analyte in the vicinity of the membrane resulted in a 200-fold enhanced ELISA signal, whereas the background signal increased only two-fold. Furthermore, only 5 μL of the sample was needed compared to 100 μL in ELISA. In the following paragraphs, we will introduce some examples of μTAS used for single and multiplex detection of bacterial toxins.

**Figure 4:**
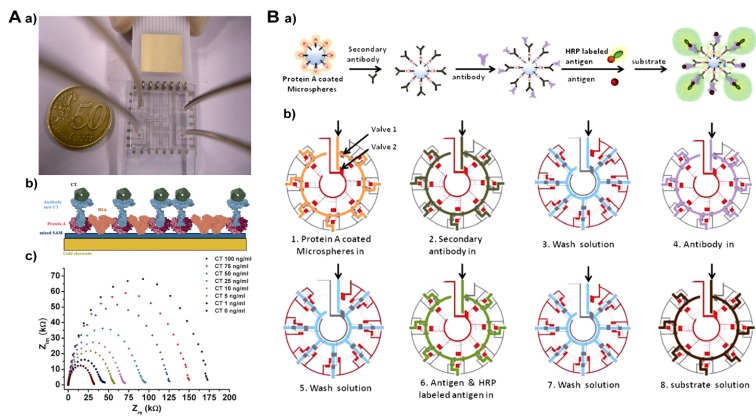
(**A**) (**a**) Biochip layout; (**b**) scheme of electrode functionalization; and (**c**) Nyquist spectra of different cholera toxin (CT) concentrations. (**B**) (**a**) Scheme of the competitive immunoassay in the immune-reaction columns; and (**b**) an illustration of the chip operations for the immunoassay. Reproduced from [102,103] with permission from The Royal Society of Chemistry.

An eight channel chip and an SWNT-based immunoassay were combined for the detection of SEB [100]. Rabbit anti-SEB antibodies are immobilized on SWNTs. The signal generated by enhanced chemiluminescence enabled the detection of 0.1 ng mL^−^^1^ of SEB in a 10-μL sample. Later, this microfluidic platform was improved for the label-free detection of SEB and termed “biological semiconductor (BSC)” [101]. A label-free impedimetric immunosensor combined with a microfluidic chip has been constructed for the B subunit of CT [102]. The increase of impedance is directly related to the bound toxin on the surface electrode, and as low as 1 ng mL^−^^1 ^of toxin can be detected (Figure 4A).

For multiplex bacterial toxin detection [24], an antibody-based microarray chip has been proposed for electrochemical detection of *Yersinia pestis*, *Bacillus anthracis* and SEB using a super avidin-biotin system [104]. In a new assay for distinguishing and quantifying botulinum neurotoxin type A (BoNT/A), SEB and the plant toxin, ricin, capture antibodies are covalently immunomobilized (printed) by a non-contact microdispensing array printer on a microstructured polymer slide serving also as an incubation chamber. Toxins are detected by biotinylated antibodies and Cy5-labeled streptavidin as the fluorescent probe [105]. Under optimized conditions, 0.5–1.0 ng mL^−^^1^ of toxins could be detected in raw milk samples. From the general layout, this chip can be used for the simultaneous detection of up to 28 analytes with six replicates. Another broadly applicable system, an immuno-column microfluidic chip, has been designed and fabricated for bacterial and algal toxin analysis, including microcystin-LR, saxitoxin and cylindrospermopsin [103]. The core principle of this new chip is the competitive immunoassay format applied in seven immuno-columns. Each column is filled with Protein A coated microspheres as the general binding support of the specific antibodies. Different primary anti-toxin antibodies are bound to the surface of the microspheres, and free (sample) toxins and HRP-labeled toxins compete for the antibody binding sites (Figure 4B). The linear range and limit of detection are 0–5.0 ng mL^−^^1^ and 0.02 ng mL^−^^1^, respectively. Recently, immunomagnetic separation in a microfluidic system and a microflow cytometer have been integrated for the automated and multiplex analysis of pathogenic bacteria [106]. Because this system is of general applicability, we present a short description. Different antibodies coupled to the surface of supraparamagnetic beads, samples, biotinylated antibodies and streptavidin labeled with phycoerythrin are pumped sequentially through and concentrated in microchannels. The fluorescence signal is generated by combinations of streptavidin phycoerythrin with two fluorophores. This automated “sample-in and answer-out” operation can be performed in less than 20 min. Another advantage of this platform is that it possesses the potential to concentrate targets from large volumes, which could improve the detection of low concentrations of toxin.

## 5. New Materials and Methods

This section will be focused on new materials and methods for signal (readout) generation, the improvement of assay performance and statistics. To increase the sensitivity of immunoassays and to reduce sample handling and processing steps is still a big challenge, and a variety of new materials and assays have been tried for their suitability to fulfill these requirements.

New nanomaterials, such as antibody-coated microspheres, have been used for the simultaneous detection of CT, SEB and other target analytes in spiked clinical samples by a microflow cytometer [107,108]. Antibodies conjugated to silica-based nanomaterials, such as fluorescent silica NPs and mesoporous silica, have been broadly applied for the detection of bacterial targets and for the enhanced loading of enzymes or other probes [55,109,110]. It should be emphasized that although the different nanomaterials are discussed separately in this review, the integration of two or more kinds of nanomaterials in one assay is becoming more and more popular, due to their complementary properties [111,112].

**Figure 5:**
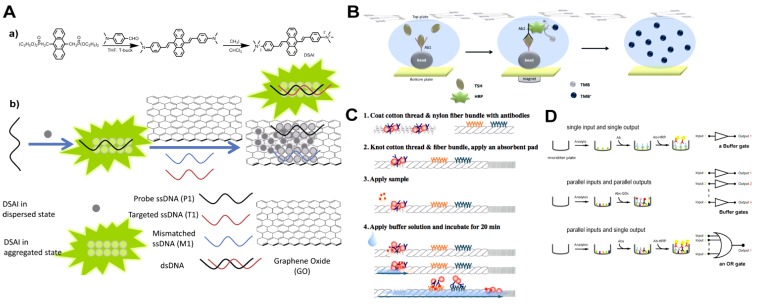
(**A**) (**a**) Synthesis of a novel aggregation-induced emission (AIE) probe (9,10-distyrylanthracene with two ammonium group, DSAI); and (**b**) a schematic description of a selective fluorescent aptasensor based on the DSAI/GO probe. Reprinted with permission from [115]. Copyright 2014 American Chemical Society; (**B**) Scheme of the key steps in the digital microfluidic (DMF) electroimmunoassay. Reproduced from [119] with permission from The Royal Society of Chemistry; (**C**) The immunochromatographic assay on a thread device assembly and assay protocol. Reprinted with permission from [120]. Copyright 2012 American Chemical Society; (**D**) The main types of assays for the detection of bacterial toxins in microtiter plates. Reproduced from [121] with permission from The Royal Society of Chemistry. ELISA for a single analyte; parallel detection of multiple analytes with multiple outputs; a single output for the detection of multiple analytes.

The pursuit of new fluorescent probes for potential applications in biological fields has created a series of enhanced materials. Among these, aggregation-induced emission (AIE) fluorescent probes have been developed for the detection of small molecules, DNA and proteins [113,114]. The AIE phenomenon shows fluorescence turn-on responses in the aggregate state in contrast to the common aggregation-caused quenching (ACQ) of fluorescent probes and offers higher sensitivity and accuracy [114]. The combination of AIE probes with nanomaterials, such as GO, and mesoporous materials show excellent performance for sensing specific targets [115,116]. For example, AIE-based aptasensors using aptamers as recognition elements and GO as efficiently adsorptive platforms have been constructed for the detection of targeted DNA and thrombin [115] (Figure 5A). In the presence of complementary single-stranded DNA (ssDNA), the formation of double-stranded DNA from the ssDNA aptamers will result in a reduced FRET effect between GO and the AIE probe-ssDNA aptamer. Therefore, the fluorescence of the AIE probe bound to dsDNA will be enhanced gradually. Similarly, conjugation of AIE probes with specific antibodies and various nanomaterials can offer new opportunities for sensitive and selective immunoassays to detect different bacterial toxins.

Metal-organic frameworks (MOFs), a class of microporous materials, offer unique properties, such as a high loading capacity, structural and chemical diversity and biodegradability, and have been used as molecular recognition elements for diverse analytical applications [117]. Particularly, the development of nanoscale MOFs (NMOFs) exhibits enormous potential, such as serving as nanocarriers for biomedical imaging and drug delivery [118]. We strongly believe that MOFs or NMOFs functionalized with antibodies will significantly improve sample enrichment and may even facilitate the development of more efficient assay systems.

Compared to immunoassays performed in microfluidic channels, digital microfluidic (DMF) systems are growing in popularity, as these approaches offer alternative open surface droplet-based fluid handling formats. In DMF systems, fluidics is electrostatically controlled in discrete droplets, in the picoliter to microliter range, on an open array of hydrophobic insulator-coated electrodes [122,123]. Separated droplets can be manipulated to mix, split and dispense from different reservoirs, eliminating the need of an oil carrier fluid [122]. These advantages make DMF suitable for both competitive and noncompetitive immunoassays. An automated DMF system was constructed to complete an immunoassay from sample application to optical readout with minimal manual intervention [123]. A three-level factorial design of experiment (DOE) was implemented to optimize the concentration and volume of the sample and incubation time for increased sensitivity in a sandwich assay. Meanwhile, a compact DMF platform integrating immunoassay and electrochemical detection has been developed [119]. Primary antibody conjugated magnetic microparticles are used for target capture, and HRP-labeled secondary antibodies catalyze the substrate for amperometrical measurement (Figure 5B).

Dipsticks and lateral flow chromatographic immunoassays, such as the pregnancy test, are well-known examples of paper-based diagnostic devices [124]. Recently, paper has been introduced as a substrate to construct microfluidic devices for rapid diagnostic tests, as “microfluidic paper analytical device (μPAD)”. μPADs offer several advantages over the widely-used polymer polydimethylsiloxane (PDMS) or poly(methyl methacrylate) (PMMA)-based sticks [125]. They can be easily designed and fabricated by using hydrophobic barriers (wax or air) and are compatible with hydrophilic solutions and small volumes of sample. μPADs provide assay times of a few minutes and require nearly no external equipment. These advantages make μPADs suitable for point-of-care testings (POCTs) and applications in resource-poor situations. In addition, other easily available materials, such as thread and polypropylene, have been used as promising supports for immunoassays to detect multiple proteins [120,126]. The sandwich immunochromatographic assay on thread emulates the principle of a lateral flow chromatographic immunoassay for a single target [120], as shown in the scheme of Figure 5C. The detection limit is in the picomolar range, and the assay performance is similar to that of paper-based and conventional assays. Furthermore, this approach has been applied for the simultaneous detection of three target proteins using three knotted threads coated with various antibodies of different specificities [120].

A promising approach in label-free technologies is the introduction of organic field-effect transistors (OFET), which enable sensitive and selective electronic applications particularly suited for POCTs [127]. OFET sensors are mostly constructed by coating aromatic or otherwise π-electron conjugated organic semiconductors with biological recognition elements, such as antibodies or DNA. One example for the detection of pathogenic *E. coli* [77] has already been mentioned. Another sensitive and selective OFET immunosensor was developed by directly monitoring the current or conductance changes in OFET to detect *E. coli* at concentrations as low as 10 CFU mL^−^^1^ [128]. In our opinion, systems integrating MOF for sample separation and OFET as the readout technique have the potential to speed up the development of sensitive and miniaturized biosensors for the simultaneous detection of different bacterial toxins.

One attractive trend in bacterial toxin analysis is the development of multiplexed analytical methods, for which some examples have been presented. These assays can be categorized into the following groups: (1) spectrally resolved assays, such as immunoassays utilizing different QDs or enzymes generating distinguishable signals; (2) spatially resolved assays, such as SPR and microarrays; (3) temporally resolved assays, such as HPLC; and (4) molecule resolved assays, such as MALDI-TOF mass spectrometry. The mathematical theories behind these assays fall into the one-to-one model (M = N), where M means the multiplexing factor and N means the number of targets, as shown in Figure 5D. However, recently, Boolean logic has been introduced for bioanalytical applications. Multiple signals can be processed by Boolean logic biosensors, which contain networks of coupled parallel biochemical reactions, to generate simple “yes or no” answers [129]. As a recent example, an OR gate-based immunoassay was developed for the simultaneous detection of seven protein components of *B. cereus* toxins [120]. In addition, a novel mathematical theory has been proposed to encode and decode multiplexed assays, which enables a theoretically unlimited number of independent targets to be detected and identified in any combination in the same sample [130].

## 6. Conclusions

The detection of bacterial toxins by antibodies has been a successful approach for decades. Future assays can hardly be imagined without the use of antibodies as recognition elements. Recent progresses in antibody engineering will further support this progress and facilitate the integration of antibodies or antibody fragments into new assay formats provided by the recent achievements in materials science. Nanomaterials, such as AuNPs, MNPs, QDs, carbon nanomaterials and MOFs, have the potential to improve sample separation, target recognition and signal amplification. The integration of these new materials into conventional immunoassays represents a promising, alternative solution to improve analytical performance. Furthermore, μTAS and microfluidic devices perform sophisticated functions, such as sample separation, signal amplification and detection, and enable “sample-in and answer-out” platforms at the centimeter scale. With these recent advances, all the tools are at hand to realize more robust, rapid and easy to operate immunoassays for the sensitive, high-throughput detection of single and multiple bacterial toxins in the near future.
